# A Machine Learning Approach to Identify Key Residues Involved in Protein–Protein Interactions Exemplified with SARS-CoV-2 Variants

**DOI:** 10.3390/ijms25126535

**Published:** 2024-06-13

**Authors:** Léopold Quitté, Mickael Leclercq, Julien Prunier, Marie-Pier Scott-Boyer, Gautier Moroy, Arnaud Droit

**Affiliations:** 1 Centre de Recherche du CHU de Québec, Université Laval, Québec, QC G1V 0A6, Canada; leopold.quitte@crchudequebec.ulaval.ca (L.Q.); mickael.leclercq@crchudequebec.ulaval.ca (M.L.); julien.prunier@crchudequebec.ulaval.ca (J.P.); mariepier.scottboyer@crchudequebec.ulaval.ca (M.-P.S.-B.); 2 Université Paris Cité, CNRS, INSERM, Unité de Biologie Fonctionnelle et Adaptative, F-75013 Paris, France; 3 Département de Médecine Moléculaire, Université Laval, Québec, QC G1V 0A6, Canada

**Keywords:** machine learning, molecular dynamics simulation, MM-PBSA analysis, protein–protein interaction, COVID-19

## Abstract

Human infection with the coronavirus disease 2019 (COVID-19) is mediated by the binding of the spike protein of the severe acute respiratory syndrome coronavirus 2 (SARS-CoV-2) to the human angiotensin-converting enzyme 2 (ACE2). The frequent mutations in the receptor-binding domain (RBD) of the spike protein induced the emergence of variants with increased contagion and can hinder vaccine efficiency. Hence, it is crucial to better understand the binding mechanisms of variant RBDs to human ACE2 and develop efficient methods to characterize this interaction. In this work, we present an approach that uses machine learning to analyze the molecular dynamics simulations of RBD variant trajectories bound to ACE2. Along with the binding free energy calculation, this method was used to characterize the major differences in ACE2-binding capacity of three SARS-CoV-2 RBD variants—namely the original Wuhan strain, Omicron BA.1, and the more recent Omicron BA.5 sublineages. Our analyses assessed the differences in binding free energy and shed light on how it affects the infectious rates of different variants. Furthermore, this approach successfully characterized key binding interactions and could be deployed as an efficient tool to predict different binding inhibitors to pave the way for new preventive and therapeutic strategies.

## 1. Introduction

The pandemic caused by the severe acute respiratory syndrome coronavirus 2 (SARS-CoV-2) was a global crisis that impacted many sectors of society, such as public health, social services, and the economy [1,2]. Since the beginning of the global pandemic in March 2020, more than 774.4 million people have been infected and over 7.0 million people have died because of this disease [3]. The binding of the SARS-CoV-2 spike protein to the human angiotensin-converting enzyme 2 (ACE2) is the first key step in the process of cell entry by the virus [4,5]. The transmembrane spike glycoprotein of the SARS-CoV-2 is composed of three heterodimers, including two subunits. The S1 subunit comprises the receptor-binding domain (RBD) that binds to ACE2, whereas the S2 subunit contains the membrane fusion machinery that allows the virus to enter the host cell [6,7]. As such, the infectivity of the virus appears to be proportional to the binding affinity of the spike protein RBD for ACE2 [6,7,8]. Thus, the RBD represents a major target for host immune surveillance and human therapies such as vaccine and inhibitor strategies [9,10,11]. On the other hand, due to the continuous transmission of the virus and despite the vaccination strategies, many variants of concern have emerged during the global spread of the disease. These variants are associated with higher transmissibility, greater virulence, and the ability to evade vaccine-mediated immunity [12,13,14]. Thus, some of these variants have caused new waves of infection in the global population, contributing to the maintenance of the pandemic. Five notable variants have been reported by the World Health Organization (WHO), including the now well-known Alpha, Beta, Gamma, Delta, and Omicron sublineages [15]. One of the most widely spread variants belonging to the Omicron sublineage was the BA.1 variant, which was later replaced by the BA.5 variants. They represented 76.2% of the sequences submitted to the GISAID initiative in October 2022 [16]. The sequence identity between the two Omicron RBDs (BA.1 and BA.5) and the Wuhan RBD is, respectively, 85.6% and 91.2%, with significant mutations located in the interaction hotspot with ACE2 [17]. The Omicron BA.1 RBD (RBD^O1^) presents a total of 22 amino acid mutations compared to the Wuhan RBD (RBD^W^) [18], while the Omicron BA.4/5 RBD (RBD^O5^) presents 17 amino acid mutations compared to the RBD^W^ [19]. Because of the rapid emergence of these new variants, it is important to analyze the impact of these mutations upon the binding modes of SARS-CoV-2 and thus develop efficient therapies to fight the disease.

Structural biology and molecular dynamics (MD) simulations have proven to be efficient tools to investigate the interaction between two protein partners [20,21]. While structure biology is necessary to experimentally determine the structure of proteins of interest and the interactions between a protein and a partner [22], MD simulations complete those analyses by allowing investigation of the dynamic properties of the complexes. The variety of structural conformations, along with the dynamic information contained within these complexes, offers a more realistic view of the behavior of protein complexes. Indeed, MD simulations have been widely used and are efficient for understanding various molecular mechanisms [23]. Due to major improvements in hardware capacity, MD simulations have become much faster in terms of computational time and can now simulate increasingly large systems over long time-scales, producing vast amounts of data [24]. However, the analysis and interpretation of these data pose a serious challenge. Several techniques have been developed to characterize the binding of two proteins, such as molecular mechanics/Poisson–Boltzmann surface area (MM/PBSA) and molecular mechanics/generalized Born surface area (MM/GBSA), which estimate the binding free energy for a complex and its decomposition by residue [25,26]. In the same way, free energy perturbation and thermodynamic integration are also able to assess the free energy of binding, but with better agreement with the experimental data [27]. Nevertheless, these methods remain computationally expensive and are less accurate in the case of charged systems [27]. Thus, the development of new efficient methods providing complementary analyses of the binding mode of a complex would favor a better understanding of molecular mechanisms.

**Figure 1:**
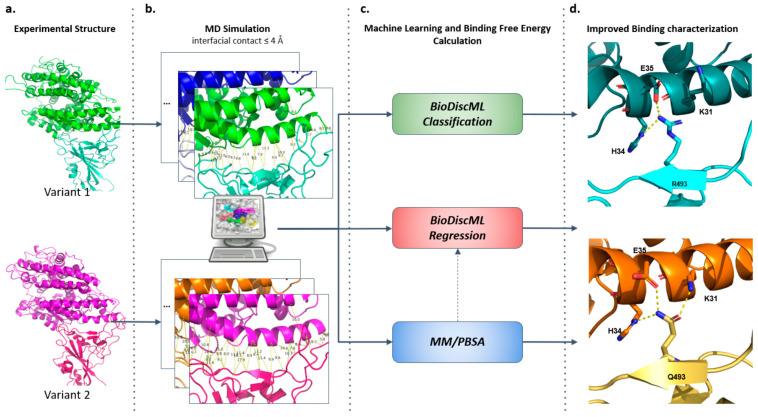
Methodology workflow. (**a**) The first step of our approach was achieve data acquisition of the variants RBD-ACE2 experimental structures in the Protein Data Bank [22]. (**b**) Then, we conducted MD simulations on the complexes and performed contact analysis using AMBER [30]. (**c**) energy was calculated using MM/PBSA [29] and machine learning classification on the interfacial contacts and regression on the binding free energy were performed using BioDiscML [31]. (**d**) This approach enabled us to obtain an improved binding characterization, with the identification of the key interactions and networks of interactions, and comparison of the SARS-CoV-2 variants binding mode.

In this study, we propose a novel approach based on machine learning (ML) and MD simulation trajectories of protein complexes to identify the interactions that play a crucial role in the formation of these complexes. To assess the relevance of this method, we decided to study the ability of RBDs from different SARS-CoV-2 variants to bind to ACE2 without considering the influence of the full spike structure. Furthermore, we did not consider the N-Glycosylation of the RBD, since carbohydrates bound to N-Glycosites seem to be only involved in the protection of the RBD in its inactive state and have no direct interaction with ACE2 [18,19,28]. We thus performed a large-scale automatic feature selection to assess the key interactions between ACE2 and three SARS-CoV-2 strains: Wuhan, Omicron BA.1, and Omicron BA.4/5. Furthermore, as we aimed to identify the interactions that differ the most in terms of binding energy and the key residues for the binding, we calculated the binding free energy of our RBDs/ACE2 complexes along with the per-residue decomposition energy [29] and trained ML models to perform regressions between the interfacial contact and the binding free energy of these complexes (Figure 1).

## 2. Results

The structures of the three complexes (ACE2-RBD^W^, ACE2-RBD^O1^, and ACE2-RBD^O5^) remained stable along the three simulations per complex (Figure S1). The average RMSD over all the replicated simulations is 2.5 Å for the complex ACE2-RBD^W^, 2.5 Å for the complex ACE2-RBD^O1^, and 2.7 Å for the complex ACE2-RBD^O5^. Most of these structural deviations were due to loop fluctuations of ACE2, especially the loops involving the residues 130–145, 336–340 or 425–430 (Figure S2), which are located far from the interacting residues (for ACE2: Q24, T27, F28, D30, K31, H34, E35, E37, D38, Y41, Q42, L79, M82, Y83, N330, K353, G354, D355, R357, and R393; for RBD: K417, G446, Y449, Y453, L455, F456, A475, F486, N487, Y489, Q493, G496, Q498, T500, N501, G502, and Y505).

### 2.1. Machine Learning Classification

To gain more insights regarding the dynamic differences of the interacting residues between the three studied complexes, we selected all pairs of residues between the RBD and ACE2 that were present at least once in the simulation at a distance lower than 4.0 Å. The contact analysis [32] was based on 3 × 3 independent simulations and resulted in the selection of 202 interactions. A total of 2079 classification models were tested to predict the strain of the SARS-CoV-2 RBD based on distances for these 202 interactions binding RBD to ACE2. A total of 706 models presented a near-perfect prediction (average MCC ≥ 0.99) with high robustness across various resamplings (standard deviation calculated on the average of MCCs across resamplings ≤ 0.01). Among them, various types of models were generated and presented excellent performances, such as K-nearest neighbors classifiers, Linear Predictive models, Random Forest algorithms, Naive Bayes algorithms, and Hoeffding Tree. Regarding the performance of the method, an average of 22 models per hour was produced using 10 CPUs.

**Figure 2:**
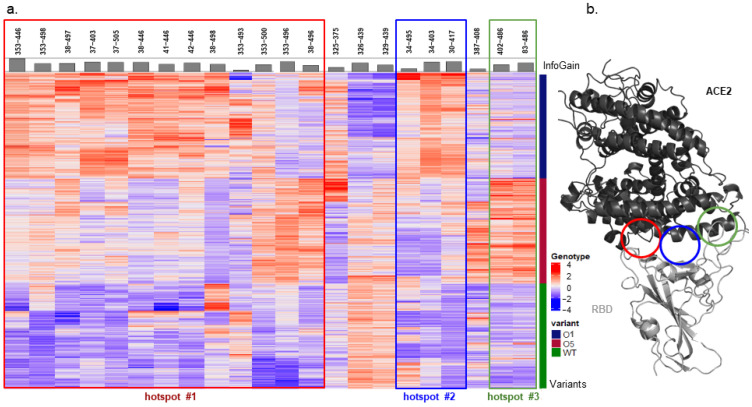
(**a**) Heatmap of the selected interfacial distances. (**b**) Hotspot of interaction localization at the interface between ACE2 and RBD. In panel (**a**) the heatmap of the selected interfacial distances (columns) of the three complexes, distance values were normalized using the *scale* function in R (z-score). Positive values were considered as long distance (red) and negative as short distance (blue). On the right side of the heatmap, the blue part of the bar represents the ACE2-RBD^O1^ distances, the red part of the bar represents the ACE2-RBD^O5^ distances, and the green part represents the ACE2-RBD^W^ distances. At the top of the heatmap, the histogram represents the information gain of the feature. The colored rectangles indicate Hotspot 1 (red), Hotspot 2 (blue) and Hotspot 3 (green). In (**b**) the structure of the complex ACE2-RBD^W^ is depicted in a cartoon representation. The colored circle locates the hotspots of interaction on the structure, Hotspot 1 (red), Hotspot 2 (blue), and Hotspot 3 (green). Figures were generated using BioDisc-Viz version 1.8.11 [35] and PyMOL [36].

Once ML calculations were completed, we selected the best model that had the lowest number of features. This model presents a MCC > 0.99 on the test set and uses 30 distances that have been reduced to 22 distances after filtering for Information Gain > 0.2, which did not reduce the model performances. The three main hotspots of interactions [33,34], as documented in the literature, were highlighted by this method (Figure 2). However, most of the selected geometric differences between the three complexes appeared to be located in a hotspot of interaction centered around K353 of ACE2 (Hotspot 1). For this hotspot and for the one located around E35 of ACE2 (Hotspot 2), we observed a similar pattern between the three complexes. Indeed, the interfacial distances of these hotspots were wide for the complex ACE2-RBD^O1^, medium for the complex ACE2-RBD^O5^, and short for the complex ACE2-RBD^W^. For the hotspot of interaction located around Y83 of ACE2 (Hotspot 3) the pattern differed. The interfacial distances were wider for the complex ACE2-RBD^O5^, and medium for the two others.

### 2.2. Binding Free Energy Calculations

**Figure 3:**
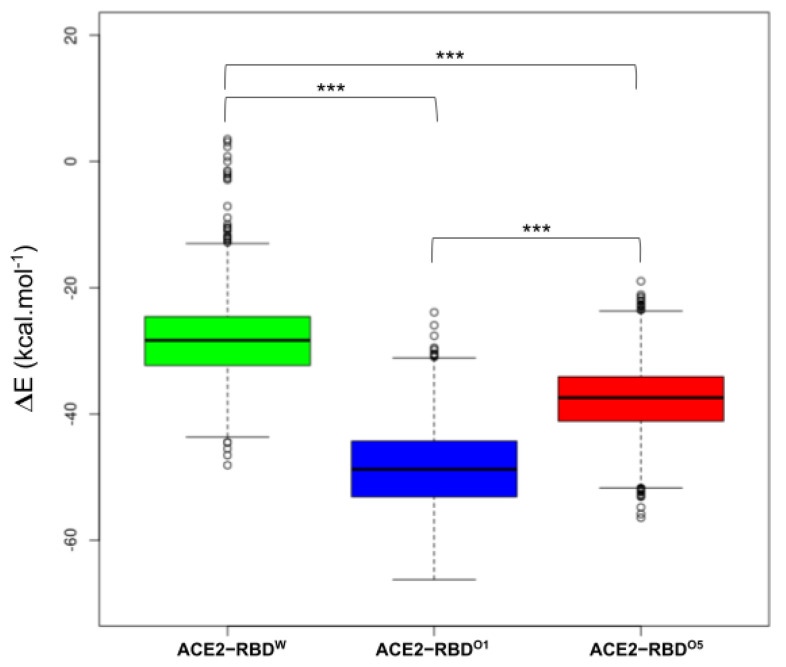
Binding free energy of ACE2 and the RBD of the SARS-CoV-2 variants. The binding free energy of the complexes was estimated in kcal.mol^−1^. In green, the boxplot of the binding free energy of the complex ACE2-RBD^W^; in blue, the boxplot of the binding free energy of the complex ACE2-RBD^O1^; in red, the boxplot of the binding free energy of the complex ACE2-RBD^O5^. *** *p*-value is less than 0.005.

To assess the impact of these selected residues in terms of binding free energy and evaluate their implication in the affinity between ACE2 and the RBD, we estimate the energy terms of the three complexes using the tool MMPBSA.py of AMBER20 (Figure 3). The difference in binding free energy between each complex was tested using Student’s t-tests (with a Bonferroni correction to take into account multiple testing) were performed to assess the difference of binding free energy (3000 frames per complex were used in the calculation). The average binding free energy of the ACE2-RBD^W^ was −28.3 (±5.9) kcal.mol^−1^, −48.6 (±6.4) kcal.mol^−1^ for ACE2-RBD^O1^ and −37.6 (±5.4) kcal.mol^−1^ for ACE2-RBD^O5^. The adjusted *p*-values associated with the differences between complexes were inferior to 2.2 × 10^−16^. These results agreed with experimental studies [37] showing an affinity of both RBD Omicron variants superior to that of the Wuhan strain, while the affinity of the Omicron BA.4/5 RBD was lower than that of the Omicron BA.1 RBD for ACE2. Hence, the observed higher infectivity of the Omicron variants compared to the Wuhan strain might be partially due to the lower free binding energy of the RBDs of the Omicrons variants compared to that of the Wuhan strain.

### 2.3. Machine Learning Regression

The BioDiscML tool was also used to train regression models between the previously extracted pairs of residues (<4.0 Å) and the associated binding free energy of the complexes to identify the key residues. The binding free energy was retrieved for every frame used in the contact analysis (MM/PBSA calculations) and used as new output. As for the previous classification analysis, the training set included 202 interactions selected over 300 frames per complex, along with the associated binding free energy. The test set was obtained in the same way but from 3000 frames. Exactly 635 models were tested on the training set. Among them, 65 presented an average Correlation Coefficient (CC) superior to 0.85 with an average Mean Absolute Error (MAE) inferior to 5.3, which was below the standard deviation of the estimated binding free energy of our complexes and thus allowed us to efficiently discriminate the complexes. Then, a consensus signature made of 30 simulated interfacial distances was extracted using the features selected by the models.

### 2.4. Residues Selection

The residues selected from our three analyses (Tables S1 and S2) were further analyzed, and a subset of 26 residues was delineated using the per-residue decomposition of the free energy provided by MMPBSA.py, along with a visualization analysis using PyMOL. The residues were closely located at the binding interface, presenting interactions and significant differences in terms of free energy per residue between the complexes (Table 1 and Table 2). We also selected some residues that had important roles in a network of interactions including strong interactions. Eleven residues of the RBDs and fifteen residues of ACE2 were selected. Only three of the eleven selected residues of the RBDs were not mutated in any SARS-CoV-2 strain, which supported the capacity of our method to retrieve the key residues involved in the variation of the binding mechanisms of similar complexes. Indeed, we expected to find mutated residues between variants responsible for major differences in their binding mode with ACE2.

### 2.5. Binding Characterization

To further investigate the dynamic differences in the interactions between the three complexes and assess the role of the selected residues, we visualized the local interactions of the hotspots using PyMOL. Hydrogen bond analysis was performed using the cpptraj tool of AMBER20. In the following section, the hydrogen bond analysis results, which are the percentages of hydrogen bonds present in the MD simulations, are presented in brackets after the residues.

**Figure 4:**
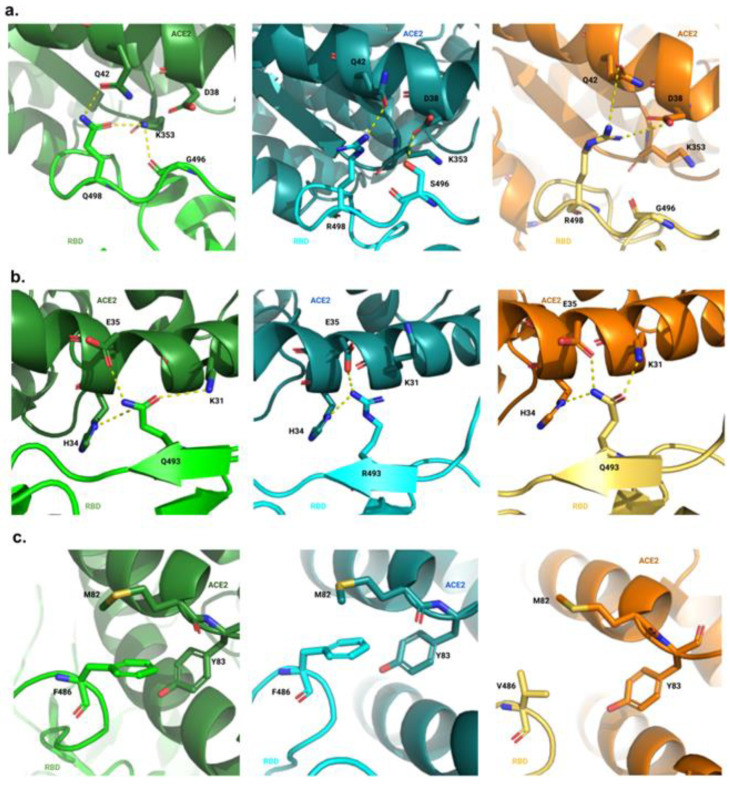
Comparison of the local interactions. In (**a**), we depict the local interactions of the hotspot centered around K353 of ACE2 (Hotspot 1). In (**b**), we depict the local interactions of the hotspot centered around E35 of ACE2 (Hotspot 2). In (**c**), we depict the local interactions of the hotspot centered around Y83 of ACE2 (Hotspot 3). In the left panel, the RBD^W^ is shown in green and the associated ACE2 structures are shown in dark green. On the middle panel, the RBD^O1^ is shown in cyan and the ACE2 structures are shown in dark cyan. On the right panel, the RBD^O5^ is shown in yellow and the ACE2 structures are shown in orange. The complexes were visualized in PyMOL using the cartoon and stick representations. Not every residue is represented to keep the visual representations as clear as possible.

For the hotspot of interaction centered around K353 of ACE2, major differences between the complexes were revealed (Figure 4a). For the complex ACE2-RBD^W^, we observed multiple hydrogen bonds: Q498-Q42 (53.0%), Q498-K353 (57.0%) and G496-K353 (43.0%). In the complex ACE2-RBD^O1^, it seemed that the Q498R mutation, along with the N501Y and G496S mutations, reorganized the network of interactions of this hotspot. The mutation N501Y produced hydrophobic repulsion on K353, which permitted two hydrogen bonds, R498-Q42 (24.0%) and S496-D38 (56.0%), and one salt bridge R498-D38. For the complex ACE2-RBD^O5^, the reversion S496G, among other structural variations, allows a strong hydrogen bond between R498 and D38 (84.0%). Near this hotspot, our method also highlighted the network of interactions around T500. For the complex ACE2-RBD^W^, we observed a strong interaction between T500-Y41 (54.0%) and T500-D337 (54.0%), along with a weaker interaction between T500-N330 (3.0%). We retrieved this last interaction according to our hydrogen bond analysis and by observing the near environment of T500 because the residue D337 was not selected with our method. For the complex ACE2-RBD^O^, the interaction T500-Y41 (47.0%) seemed weaker than in previous observations, while T500-D337 (54.0%) remained stable. This may be due to a stronger interaction between T500 and N330 (8.0%). For the complex ACE2-RBD^O5^, the interaction T500-Y41 (44.0%) was weaker, but with a stronger interaction between T500 and D337 (65.0%). The T500-N330 interaction (7.0%) remained stable compared with the complex ACE2-RBD^O^.

For the second hotspot located around E35 of ACE2, four residues were selected using our approach (Figure 4b). In the ACE2-RBD^W^ complex, the residue Q493 presented three hydrogen bonds with the residues H34 (14.0%), E35 (60.0%), and K31 (15.0%). In this hotspot, the interaction Q493-E35 was retrieved in accordance with the hydrogen bond analysis and by observing the nearby environment of Q493R because the residue E35 was not selected. Nevertheless, except for this bond with E35, the interactions formed between Q493, and the other two residues seemed to vary between the complexes. Indeed, the interactions that formed between the residues R493 with H34 (40.0%) and E35 (82.0%) in the ACE2-RBD^O1^ complex were much more stable, even though only two of the three previous residues participated in these interactions. Moreover, in the ACE2-RBD^O5^ complex, the network of interactions, including the reverse mutation R493Q, was stabilized by the loss of interaction between E484A-K31. We observed a more stable interaction between Q493 and E35 (64.0%) and K31 (43.0%).

For the third hotspot of interaction around Y83 of ACE2, five residues were highlighted using our method (Figure 4c). For the complexes ACE2-RBD^W^ and ACE2-RBD^O^, we observed a pi-stacking interaction between Y83 of ACE2 and F486 of the two RBD. We also assumed a hydrophobic interaction between M82 and F486 that stabilized the previously cited interaction (F486-Y83). Furthermore, we observed two interactions between N487 with Y83 (79.0%) and Q24 (27.0%) for the ACE2-RBD^W^ complex, and with Y83 (85.0%) and Q24 (15.0%) for the ACE2-RBD^O^ complex. On the other hand, the mutation F486V in the RBD^O5^ canceled the interactions of F486V-Y83 and F486V-M82, while the interaction of N487-Y83 (72.0%) was maintained and the interaction of M487-Q24 (4.0%) was reduced. According to Cao et al., the mutation F486V would benefit the Omicron BA.5 sublineage in terms of immune escape, while the F486 residue enhanced the binding affinity of the RBDs for ACE2 [37].

All these differences in the binding mode help us to explain the difference in terms of binding free energy between the three complexes and, thus, the difference in terms of infectivity of these three SARS-CoV-2 strains.

## 3. Discussion and Conclusions

In this study, we showed the power of using machine learning classification and regression to explore and analyze the binding mode and the impact of genetic variants for a protein complex illustrated with SARS-CoV-2 RBD and ACE2. The results were consistent with those of previous studies, including experimental ones [34,37,38]. In the pandemic context caused by this kind of rapidly mutating virus, this approach would fulfil the need for accurate and fast analyses that would permit us to efficiently understand the binding mode of the variants to their target while also allowing us to identify the key interactions.

The machine-learning approach was able to highlight three hotspots of interactions and select the residues that likely play a key role in the interaction of RBD with ACE2. In addition to the binding free energy calculations, this approach can also be used as a complementary analysis that allows us to efficiently characterize the binding interface and assess the impact of the residues in terms of binding free energy. Furthermore, this framework allows us to identify key interacting residues and networks of interactions which could help us to develop inhibitor strategies. Altogether, the main advantages of this method can be described as follows. (1) The methodology efficiently exploits the vast amount of data produced by the MD simulations. (2) It works without bias, as the input dataset is only constructed on the distances between interfacial residues that form a contact during the MD simulation (the threshold to determine the contacts can be adapted depending on the studied systems). (3) Using the binding free energy calculation enabled us to focus only on the role of the interfacial residues and thus to focus our analysis. (4) Finally, this method enables the user to highlight the role of specific residues that could not have been significantly detected by the energy decomposition analysis provided by MM/PBSA calculations. For example, some RBD residues, such as N487, T500 or wild type G496, did not present absolute high energy or differences between the variants but were selected by our method and seem to play a key role in the binding of the RBDs to ACE2. It appears that these residues have significant roles in networks of interactions at the interface and, as such, our approach allows us to identify these networks and key interactions.

However, this approach exhibits several limitations. It relies on the MD simulation methodology, and thereby is contingent upon the quality of the initial model or the precision of the utilized force field [39]. Moreover, to select the key interfacial residues in terms of binding free energy, the method requires other tools to calculate the binding free energy. The analysis of the results still requires the attention of an expert on the selected interactions to interpret and assess the predictions of the produced models, but significantly accelerates the analysis of biomolecular systems.

The choice of approach was informed by the complexity of the classification problem and the extensive number of geometry features involved. We opted for machine learning techniques, which are particularly well suited for such scenarios. This approach offers a fast and cost-effective method compared to high free energy calculations and wet lab experiments. As many models yielded accuracies and MCCs exceeding 98%, this demonstrated the general efficiency of machine learning for our specific application. This high performance of various models is linked to several factors. The data were characterized by clear patterns and a strong signal-to-noise ratio, which enhanced the effectiveness of simple models like Naive Bayes or K-nearest neighbors. Furthermore, the high performance in other methods such as tree-based and indicates that the structure of the data contains clear decision boundaries despite the non-linear data relationships between features and the classes to predict. Overall, this suggests that the results are not due to overfitting, implying that the findings are robust and generalizable and that they reflect essential patterns indicative of underlying biological realities. Finally, though this is unusual, we used a larger test set to enable a more realistic evaluation of the model’s performance, as it can be repeatedly tested on new data indefinitely. A larger test set simulates this scenario and demonstrates that the model does not overfit the training data. Additionally, this approach assesses how well the model generalizes when encountering fewer common instances.

To conclude, this novel approach successfully characterized the key binding interactions and the major differences in the binding mode of the three variants to ACE2, as previously documented in the literature. This approach and the resulting analysis can be adapted to other biomolecular systems in various contexts in the development of new therapeutic molecules, for instance. Indeed, this methodology could be employed for in silico protein design, where the comparison of similar systems, and the selection and understanding of networks and key interactions, especially in terms of binding free energy, are important steps in the process of developing new molecules.

## 4. Materials and Methods

### 4.1. Data Acquisition and Preprocessing

The experimental structures of the SARS-CoV-2 RBD variants in complexes with human ACE2 were retrieved from the website of Protein Data Bank [22] (Wuhan strain RBD -ACE2, named 6M0J [28]; Omicron BA.1 strain RBD-ACE2, named 7U0N [18]; and Omicron BA.4/5 strain RBD, named 7ZXU [19] aligned with Omicron BA.1 structure to obtain the structure for the complex with ACE2 [40]). This structural alignment was performed using PyMOL [36] with an associated root-mean-square deviation (RMSD) of 0.7 Å.

### 4.2. Molecular Dynamics Simulation

The following analyses were performed using the force field ff14sb of Amber 34. The protonation state was determined using the PDB2PQR web server [41]. We have set the physiological pH to 7.4 with the force field AMBER. The molecular system was then neutralized by the addition of counterions. To perform the simulations in an explicit solvent, the simulations were set up using the TIP3P water model [42]. The system was embedded in a rectangular box of water with a minimal distance of 10 Å between the complex and the water box borders using the tool tleap of AMBER [30]. Seven steps of minimization were applied to our systems using the program sander of AMBER20 37. In the first step, strong harmonic restraints (50 kcal.mol^−1^.Å^−2^) were carried out on the protein complex to minimize the solvent. Then, the restraints were applied only to the backbone of the protein and gradually reduced from 25 to 1 kcal.mol^−1^.Å^−2^. Finally, a final step of minimization without restraint was conducted. Each of these minimization steps consisted of 1000 iterations: 500 iterations using the steepest descent algorithm [43], and 500 iterations using the conjugate gradient method [44].

After the minimization, simulations included heating the systems from 100 K to 310 K with a constant volume and restraints on the backbone of 25 kcal.mol^−1^.Å^−2^ during 100 ps, then 4 steps of equilibration with the NPT ensemble (constant pressure and temperature) were conducted. A pressure of 1 bar and temperature of 310 K were maintained using the coupling algorithm of Berendsen [45]. These 4 steps of equilibration of 50 ps each were conducted with energy restraints on the backbone, which were gradually reduced.

Complexes of the Wuhan strain, Omicron BA.1, and Omicron BA.5 RBDs with ACE2 were simulated for 3 × 100 ns per complex using the program pmemd.CUDA of AMBER20. Simulations were conducted without restraints in an NPT ensemble with a time step of 2.0 fs.

### 4.3. Decomposition and Free Energy Binding Calculation

The free energy binding and residue contributions to this energy were calculated using the method MM/PBSA implemented in the program MMPBSA.py of AMBER20 [29]. This method is considered to be a gold standard for estimating the free energies of complexes. The binding free energy of each complex was calculated using the following equation:

ΔE = Ec − (ER + EL)

where ΔE is the binding free energy of the complex, E_c_ is the free energy of the complex, E_R_ is the free energy of the receptor, and E_L_ is the free energy of the ligand.

The free energy *E* of each state is calculated as a sum of 5 energy contributions, as follows:

E = E_VdW_ + E_Ele_ + E_PB_ + E_Pol_ + E_Disp_

where E_VdW_ is the van der Waals contribution, E_Ele_ is the electrostatic contribution, E_PB_ is the electrostatic contribution to the solvation free energy calculated using the Poisson–Boltzmann (PB) equation, E_Pol_ is the polar contribution, and E_Disp_ is the dispersion contribution.

The internal dielectric constant was set to 12 and we use 1000 conformations per replicate.

### 4.4. Machine Learning

To characterize the key interaction between the RBD of the SARS-CoV-2 variants and ACE2 using machine learning approaches, we first performed a feature extraction based on pairs of residues in contact (<4.0 Å) at the interface of the complex using the tool cpptraj [32] of AMBER20 for each replicate. Only the contacts including one residue that belonged to ACE2 and one that belonged to the RBD were selected. Contacts identified for each complex were merged, resulting in 202 residue pairs. The training set for the machine learning step included distances for the corresponding 202 residue pairs on 100 frames per simulation (300 frames per variant) which were sampled with a time step of 1 ns throughout the simulations. The test set was similarly obtained using 1000 frames per simulation (3000 per complex) sampled with a time step of 100 ps. We added the variant as an additional feature to the test and training set from which the simulation frame came and then added the associated binding free energy for each frame calculated with MM/PBSA [29]. In all cases, the datasets were equilibrated between the classes.

The models were trained to discriminate the variants, WT vs. Omicron BA.1 vs. Omicron BA.5, using the extracted features, i.e., distances between interfacial residues of ACE2 and the RBDs. We then trained multiple machine learning models using BioDiscML version 1.8.11 [31], a sequential minimal optimization algorithm for machine learning based on the Weka Java library [46,47]. This tool is able to train thousands of models by employing various classifiers with many hyperparameters combinations. Thus, a variety of machine learning models have been used for classification, including rules-based methods (i.e., OneR, PART, Prism, Ridor, and RoughSet), decision tree models (i.e., BFTree, J48, RandomForest, and DecisionStump), Bayesian models (i.e., NaiveBayes and BayesNet), lazy classifiers (i.e., KStar and LocalKnn), and function-based models (i.e., Logistic, SimpleLogistic), Multilayer Perceptron and support vector machine, among others. These various models have been tried with various sets of hyperparameters, as described in https://github.com/mickaelleclercq/BioDiscML/blob/master/classifiers.conf (accessed on 1 November 2022) (functions (linear, logistic, etc.): 28 models (25%); bayes (BayesNet, NaiveBayes): 27 models (24%); trees (J48, Random Forest, etc.): 21 models (19%); lazy: 15 models (12%); rules: 14 models (13%); miscellaneous: 8 models (7%)). The same percentages apply for all generated models, as they were executed with the same search modes, i.e., stepwise learning (forward, backward, and forward–backward) and top k features (i.e., k = 1, 5, 10, 20, 30, 40, 50, 75, 100, 150, 200), and the same optimizers (AUC, MCC, FDR, BER, ACC). Hyperparameters were pre-configured directly using BioDiscML version 1.8.11. Since near-perfect models were obtained, no hyperparameter optimization was performed. To avoid overfitting, an automated stratified sampling was performed to create a validation set and all models underwent evaluation through cross-validation methods like k-fold, bootstrapping, and repeated holdout, where the standard deviation was also determined. Furthermore, feature selection was carried out through stepwise learning in addition to the top-k features using a ranking based on information gain. This capability of selecting a combination of features with a high predictive potential is crucial here. By choosing a subset of distances to predict variants’ binding modes, this method emphasizes the major dynamic differences in how variants bind to ACE2 and identifies key residues. Then, from the many models generated, we selected the best model based on the global predictive performance revealed by the average Matthews Correlation Coefficient (MCC) based on several evaluations (10-fold cross-validation, leave-one-out cross-validation, holdout, repeated holdout, and bootstrapping) with low standard deviation across evaluation and a small number of interactions.

The second phase of our methodology involved training machine learning models to predict the binding free energy from the distances between interfacial residues of ACE2 and the RBDs. Similar to the first phase, by selecting a subset of these distances, we could pinpoint key interactions, this time focusing on variations in binding free energy between ACE2 and the variants. The most effective models were chosen based on their average Correlation Coefficient (CC) and Mean Absolute Error (MAE), following the evaluations previously described. From these top models, we extracted a consensus signature and applied it in PyMOL to explore the specific roles of the interactions.

## Figures and Tables

**Table 1:** Identified RBD residues selected by classification and regression ML methods along with the associated per-residue decomposition of the free energy. Amino acids are indicated in the following order: RBD^W^, RBD^O1^, and finally RBD^O5^. The method that selected the residue is indicated with a cross. The estimated free energy (FE) per residue and per complex are in kcal.mol^−1^. In this table, the residues that were closely located at the binding interface, presenting interactions, and with high differences in terms of free energy per residue between the complexes were retained. Some residues that seemed to have a key role in a network of interactions including strong interactions were also selected.

Amino Acid	Position	Classification	Regression	FE WT kcal.mol^−1^	FE O1 kcal.mol^−1^	FE O5 kcal.mol^−1^
R–K–R	403	x	x	−132.0	−117.5	−120.5
K–V–N	417	x		−132.7	−2.1	−1.8
E–A–A	484		x	97.0	−0.2	−0.3
F–F–V	486	x		−7.0	−6.2	−3.0
N–N–N	487		x	−6.0	−5.8	−6.0
Q–R–Q	493	x	x	−14.1	−142.2	−11.8
S–S–S	494		x	2.8	−1.8	0.5
G–S–G	496	x	x	−4.3	−4.5	1.8
Q–R–R	498	x	x	−8.8	−138.4	−153.5
T–T–T	500	x		−6.0	−6.7	−6.5
Y–H–H	505	x		−11.1	−8.3	−10.9

**Table 2:** Identified ACE2 residues selected by classification and regression ML methods along with the associated per-residue decomposition of the free energy. The method that selected the residue is indicated with a cross. The estimated free energy (FE) per residue and per complex are in kcal.mol^−1^. In this table, the residues that were closely located at the binding interface, presenting interactions, and had high differences in terms of free energy per residue between the complexes were retained. The residues with important roles in a network of interactions including strong interactions were also selected.

Amino Acid	Position	Classification	Regression	FE WT kcal.mol^−1^	FE O1 kcal.mol^−1^	FE O5 kcal.mol^−1^
Q	24		x	−5.5	−9.4	−6.7
D	30	x		−46.4	−42.2	−46.0
K	31		x	−3.7	49.0	46.3
H	34	x	x	−5.5	−9.2	−8.2
E	35		x	−17.5	−75.4	−62.5
E	37	x		−31.5	−53.6	−60.9
D	38	x	x	−24.2	−73.6	−90.5
Y	41	x	x	−3.0	−2.3	−2.3
Q	42	x	x	−4.0	−1.8	1.2
M	82	x	x	−1.5	−0.9	−0.9
Y	83	x	x	−4.8	−4.8	−2.8
N	330		x	−1.0	−1.4	−1.4
K	353	x	x	2.4	50.3	55.6
A	386		x	−1.2	−1.3	−2.3
A	387	x		−0.9	−0.7	−1.2

## Data Availability

The data are available on https://github.com/leopoldquitte/BioDiscML_MD_Analysis (accessed on 22 May 2024).

## Supplementary Materials

The following supporting information can be downloaded at: https://www.mdpi.com/article/10.3390/ijms25126535/s1.
