# Supplementary material for: A Machine Learning Approach to Identify Key Residues Involved in Protein–Protein Interactions Exemplified with SARS-CoV-2 Variants
Source: Int J Mol Sci. 2024 Jun 13;25(12):6535. doi: 10.3390/ijms25126535 (PMC11204244; doi:10.3390/ijms25126535)
Supplement: Supplementary file 1 [file ijms-25-06535-s001.zip › ijms-2977777-supplementary.pdf]

Supplementary data: A machine learning approach to identify key residues involved in protein-protein interactions exemplified with SARS-CoV-2 variants.

Léopold Quitté[1], Mickaël Leclercq[1], Julien Prunier[1], Marie-Pier Scott-Boyer[1], Gautier Moroy[2,] and Arnaud Droit[1,3,*]*

1 Centre de Recherche du CHU de Québec-Université Laval, Québec, QC, Canada.
2 Université Paris Cité, CNRS, INSERM, Unité de Biologie Fonctionnelle et Adaptative, F-75013 Paris, France.
3 Département de Médecine Moléculaire, Université Laval, Québec, QC, Canada.

* Correspondence: gautier.moroy@u-paris.fr & arnaud.droit@crchudequebec.ulaval.ca

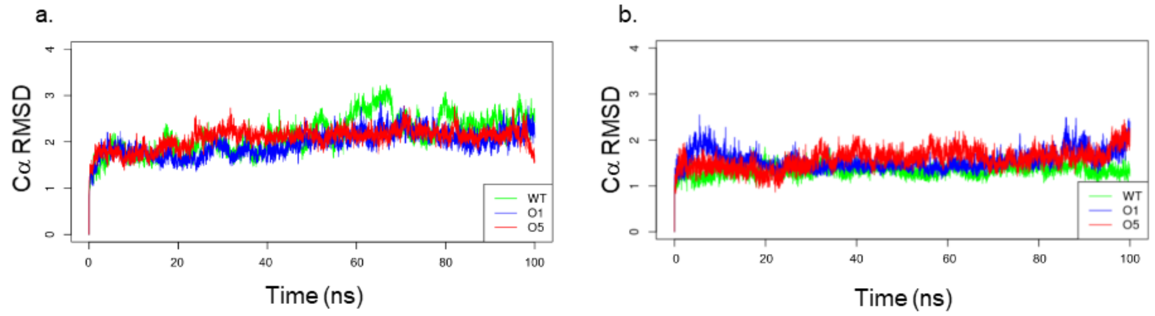

Figure S1. Root Mean Square Deviation (RMSD) of the complexes partner during the simulations. (a) RMSD of the ACE2 structures, in green bound to the RBD^{W}, in blue bound to the RBD^{O1}, in red bound to the RBD^{O5}. (b) RMSD of the RBD structures, in green the RBD^{W}, in blue the RBD^{O1}, in green the RBD^{O5}. The data shown on these figures comes from the first replicates of our simulations.

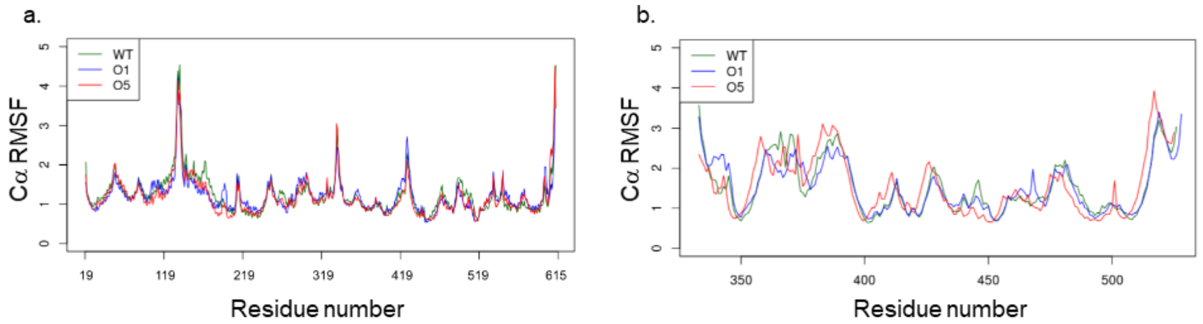

Figure S2. Root Mean Square Fluctuations (RMSF) of the complexes partner during the simulations. (a) RMSF of the ACE2 structures, in green bound to the RBD^{W}, in blue bound to the RBD^{O1}, in red bound to the RBD^{O5}. (b) RMSF of the RBD structures, in green the RBD^{W}, in blue the RBD^{O1}, in green the RBD^{O5}.

Table S1. RBD residues selected by classification and regression ML methods along with the associated per residue decomposition of the free energy. The amino-acids nature indicated order is first the one belonging to the RBD^{W}, then to RBD^{O1}, last to RBD^{O5}. The method that selected the residue is indicated with a cross. The estimated free energy (FE) per residue and per complex are in $kcal.mol^{-1}$. The data shown on these figures comes from the first replicates of our simulations. For the other replicates the results are similar.
The data shown on these figures comes from the first replicates of our simulations. For the other replicates the results are similar.

Amino Acid	Position	Classification	Regression	FE WT $kcal.mol^{-1}$	FE O1 $kcal.mol^{-1}$	FE O5 $kcal.mol^{-1}$
S – S – F	371		x	0.7	0.6	0.3
S – S – F	375	x		-0.3	-0.1	-0.2
R – K – R	403	x	x	-132.0	-117.5	-120.5
R – S – S	408	x		-112.1	-106.8	-1.3
K – V – N	417	x		-132.7	-2.1	-1.8
N – N – N	437		x	-1.9	-1.7	-1.9
N – R – N	439	x		-0.7	-132.0	-0.7
S – A – S	443		x	-1.3	-1.9	-1.8
K – T – K	444		x	-100.9	1.7	-99.8
V – S – V	445		x	-2.0	1.6	-2.0
G – T – G	446	x		-0.9	0.5	0.0
G – G – G	447		x	-1.0	-1.4	-1.3
R – R – R	454		x	-88.0	-85.0	-85.6
I – I – I	472		x	-1.2	-1.2	-1.2
A – A – A	475		x	-6.3	-3.8	-7.0
G – G – G	476		x	-1. 9	-2.3	-3.7
P – P – P	479		x	1.0	1.1	1.2
V – V – V	483		x	-0.4	-0.4	-0.4
E – A – A	484		x	97.0	-0.2	-0.3
G – G – G	485		x	-0.9	-0.9	-0.7
F – F – V	486	x		-7.0	-6.2	-4.0
N – N – N	487		x	-6.00	-5.8	-6.0
C – C – C	488		x	0.0	-0.2	0.0
P – P – P	491		x	1.3	1.4	1.2

L – L – L	492		x	-1.5	1.0	-0.1
Q – R – Q	493	x	x	-14.1	-142.2	-11.8
S – S – S	494		x	2.8	-1.9	0.5
Y – Y – Y	495	x		-2.7	-0.5	-0.7
G – S – G	496	x	x	-4.3	-4.5	1.8
F – F – F	497	x		-0.7	-0.9	-0.8
Q – R – R	498	x	x	-8.8	-138.4	-153.5
P – P – P	499		x	-0.1	0.5	-0.2
T – T – T	500	x		-6.0	-6.7	-6.5
Y – H – H	505	x		-11.1	-8.3	-10.9

Table S2. ACE2 residues selected by classification and regression ML methods along with the associated per residue decomposition of the free energy. The method that selected the residue is indicated with a cross. The estimated free energy (FE) per residue and per complex are in kcal.mol^{-1}.

Amino Acid	Position	Classification	Regression	FE WT kcal.mol^{-1}	FE O1 kcal.mol^{-1}	FE O5 kcal.mol^{-1}
I	21		x	-0.5	-1.0	-0.8
Q	24		x	-5.5	-9.4	-6.7
T	27		x	-4.3	-5.0	-4.2
D	30	x		-46.4	-42.2	-46.0
K	31		x	-3.7	49.0	46.3
H	34	x	x	-5.5	-9.2	-8.2
E	35		x	-17.5	-75.4	-62.5
E	37	x		-31.5	-53.6	-60.9
D	38	x	x	-24.2	-73.6	-90.5
Y	41	x	x	-3.0	-2.3	-2.3
Q	42	x	x	-4.0	-1.8	1.2
M	82	x	x	-1.5	-1.0	-1.0
Y	83	x	x	-4.8	-4.8	-2.8
Q	325	x	x	0.2	-0.6	-0.2
G	326	x	x	0.3	0.2	0.2
E	329	x		-15.7	-53.9	-45.3
N	330		x	-1.0	-1.4	-1.4
K	353	x	x	2.4	50.3	55.6
A	386		x	-1.2	-1.3	-2.3
A	387	x		-0.9	-0.7	-1.2
